# Microscopic polyangiitis with pulmonary involvement: Case Report

**DOI:** 10.3389/fmed.2026.1905035

**Published:** 2026-08-18

**Authors:** Miguel Ochoa-Andrade, Sofía Muñoz, Brittany González, Mateo Rojas, Tanya Mideros, José Santana, Andrea Quishpe, Javier Velasteguí, Franklin Uguña

**Affiliations:** 1 Medicine Program, Faculty of Health Sciences, Universidad Técnica Particular de Loja, Loja, Ecuador; 2 Medicine Program, Faculty of Health Sciences, Pontificia Universidad Católica del Ecuador, Quito, Ecuador; 3 Nephrology, Medical Service, General Coordination of Clinical Services, Hospital General del Sur de Quito, Quito, Ecuador; 4 Internal Medicine, Medical Service, General Coordination of Clinical Services, Hospital General del Sur de Quito, Quito, Ecuador; 5 Adult Intensive Care Service, Subdirectorate of Critical Care Medicine, Hospital General del Sur de Quito, Quito, Ecuador; 6 Rheumatology, Medical Service, General Coordination of Clinical Services, Hospital General del Sur de Quito, Quito, Ecuador

**Keywords:** antibodies, antineutrophil cytoplasmic, anti-neutrophil cytoplasmic antibody-associated Vasculitis, glomerulonephritis, lung injury, microscopic polyangiitis, mieloperoxidase, pulmonary alveoli

## Abstract

**Introduction:**

ANCA-associated vasculitis is a rare autoimmune disorder characterized by necrotizing inflammation of small vessels, frequently affecting the kidneys and lungs, and may present as a potentially life-threatening pulmonary-renal syndrome during disease relapse.

**Case presentation:**

We present the case of a 66-year-old woman with a history of MPO-ANCA-associated vasculitis with prior renal and pulmonary involvement, who was admitted with acute respiratory failure and rapidly progressed to severe acute respiratory distress syndrome requiring invasive mechanical ventilation, along with acute kidney injury (KDIGO stage 3B) superimposed on chronic kidney disease. Laboratory findings revealed progressive anemia, elevated inflammatory markers, and markedly increased MPO-ANCA titers, compatible with vasculitis reactivation presenting as diffuse alveolar hemorrhage and pulmonary-renal syndrome. The patient was treated with pulse methylprednisolone, intravenous immunoglobulin, plasmapheresis, and rituximab, in addition to intensive supportive care. Her clinical course was complicated by a urinary tract infection caused by extended-spectrum beta-lactamase (ESBL)-producing *Escherichia coli*. After gradual immunomodulatory treatment, she showed progressive recovery of respiratory and renal function, with resolution of the alveolar hemorrhage and satisfactory discharge from the intensive care unit.

**Conclusion:**

This case highlights the diagnostic challenges and severity of MPO-ANCA vasculitis relapse, and underscores the importance of early recognition, prompt immunosuppressive therapy, and multidisciplinary management to improve outcomes, especially in healthcare settings with limited resources.

## 1 Introduction

Small vessel vasculitis can be broadly classified according to its underlying pathogenic mechanism into immune complex-mediated vasculitis and antineutrophil cytoplasmic antibody (ANCA)-associated vasculitis. The latter group includes granulomatosis with polyangiitis (GPA), microscopic polyangiitis (MPA), eosinophilic granulomatosis with polyangiitis (EGPA), and pauci-immune necrotizing glomerulonephritis limited to the kidney (1, 2).

ANCA-associated vasculitis is commonly characterized by two main immunofluorescence patterns: cytoplasmic ANCA (c-ANCA), generally directed against proteinase 3 (PR3), and perinuclear ANCA (p-ANCA), usually directed against myeloperoxidase (MPO). PR3-ANCA is most frequently associated with GPA, while MPO-ANCA is more often observed in MPA and EGPA (3).

Microscopic polyangiitis is a necrotizing small-vessel vasculitis strongly associated with ANCA, particularly MPO-ANCA, and is histologically defined by a pauci-immune pattern with minimal or absent immune complex deposition. These features distinguish it from immune complex-mediated vasculitis and form the basis for understanding its clinical manifestations and pathophysiology (4).

The estimated global annual incidence ranges from 10 to 20 cases per million inhabitants. The frequency varies according to geographic region and ethnic group: in Europe, granulomatosis with polyangiitis predominates (60–70% of cases), more often associated with PR3-ANCA, while in Asian populations, microscopic polyangiitis is more common (60–80%), mainly associated with MPO-ANCA. Incidence increases with age, particularly between 65 and 74 years, and shows a slight male predominance, with an approximate male-to-female ratio of 1.5:1 (5–7).

In Latin America, epidemiological data are limited. Microscopic polyangiitis (MPA) (29–40%) occurs predominantly in adults over 50 years of age and is characterized by a high frequency of pauci-immune renal involvement and pulmonary manifestations. In healthcare settings with limited resources, restricted access to specialized immunological tests, immunosuppressive therapy, and multidisciplinary follow-up may delay timely diagnosis and treatment, increasing the risk of renal and alveolo-pulmonary complications as well as mortality rates (8, 9).

Although considered rare diseases, ANCA-associated vasculitis are an important cause of rapidly progressive glomerulonephritis and pulmonary-renal syndrome, present in approximately 30–50% of cases, and characterized by severe renal involvement and diffuse alveolar hemorrhage (10, 11). In Latin America, epidemiological data are limited; microscopic polyangiitis accounts for approximately 29–40% of reported cases and predominantly affects adults over 50 years of age, with a high frequency of pauci-immune renal involvement and pulmonary manifestations (12).

In Ecuador, there are no extensive national epidemiological records or large population series published on the incidence or prevalence of microscopic polyangiitis or ANCA-associated vasculitis in general; most available data come from case reports or small hospital center series. In healthcare settings with limited resources, restricted access to specialized immunological tests, immunosuppressive therapy, and multidisciplinary follow-up may delay timely diagnosis and treatment, thus increasing renal and alveolo-pulmonary complications as well as mortality rates. Gaps in health coverage, geographic barriers, and limitations in specialized hospital infrastructure can negatively affect the prognosis of complex multisystem diseases such as microscopic polyangiitis, perpetuating health disparities and adverse clinical outcomes (13).

## 2 Case presentation

A 66-year-old woman, residing in Quito, with a diagnosis of MPO-ANCA-associated vasculitis with pulmonary and renal involvement, had been under Rheumatology follow-up since 2024. In May of this year, she experienced an episode of diffuse alveolar hemorrhage treated with cyclophosphamide as induction therapy for 6 months, followed by rituximab (cumulative dose of 2 g) in June 2025. At her last outpatient appointment, disease activity persisted, evidenced by proteinuria of 3–4 g/24 h, for which she was receiving prednisone 20 mg/day and was scheduled for a new dose of rituximab.

In January 2026, she presented to the emergency department with profuse watery diarrhea, vomiting, and progressive dyspnea of 12 h’ duration, initially on exertion and later at rest. On admission, she showed severe hypoxemia (SpO₂ 62% on room air), tachycardia (124 bpm), and a marked increase in respiratory effort, requiring immediate orotracheal intubation due to refractory hypoxemia. Arterial blood gas analysis with FiO₂ of 1.0 revealed a PaO₂ of 58 mmHg (PaO₂/FiO₂ = 58), compatible with severe acute respiratory distress syndrome (ARDS). Initial tests showed hemoglobin of 8.9 g/dL, C-reactive protein of 186 mg/L, procalcitonin of 0.42 ng/mL, and serum creatinine of 2.19 mg/dL, which later increased to 2.61 mg/dL. Acute kidney injury was documented (KDIGO stage 3B) superimposed on chronic kidney disease, with proteinuria of 3,163 mg/24 h and preserved diuresis.

**Figure 1:**
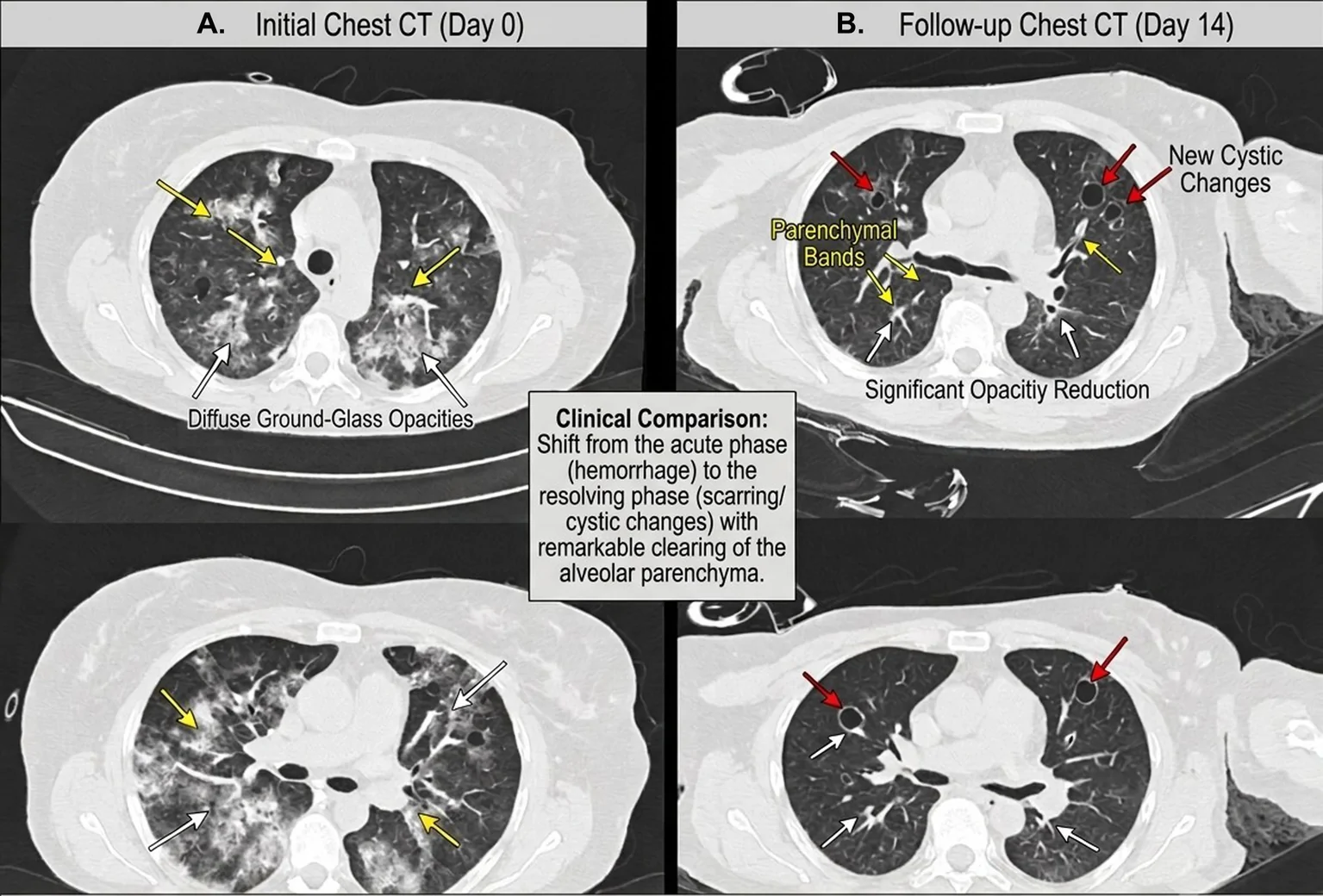
Non-contrast chest computed tomography (CT) scan—admission and control. **(A)** (Day 0): Chest CT showing diffuse ground-glass opacities and areas of hemorrhage (white). The scan shows extensive lung involvement. **(B)** (Day 14) Follow-up CT demonstrating dramatic interval reduction of ground-glass opacities. There are new findings of persistent parenchymal bands (yellow arrow) and development of pulmonary cysts (red arrows), indicating a shift from hemorrhage to scarring/organizing phase.

Chest computed tomography on admission showed bilateral ground-glass opacities and small perihilar-predominant consolidations, consistent with acute diffuse alveolar hemorrhage (Figure 1A).

## 3 Discussion

### 3.1 Pathophysiology of ANCA-associated vasculitis

The reactivation of microscopic polyangiitis with pulmonary and renal involvement represents an immunological emergency with a high short-term mortality rate if not promptly addressed. The loss of immune tolerance to myeloperoxidase induces the synthesis of pathogenic autoantibodies (MPO-ANCA), which directly interact with neutrophils primed by circulating pro-inflammatory cytokines, such as tumor necrosis factor-alpha and interleukin 1 beta (14). This interaction triggers massive degranulation, the release of reactive oxygen species, and the formation of neutrophil extracellular traps (15).

The injurious phenomenon culminates in necrotizing pulmonary capillaritis and pauci-immune crescentic glomerulonephritis. These entities are characterized by generalized endothelial denudation and destructive inflammation of small-caliber blood vessels without significant immune complex deposition (14, 15). Rapid mechanical removal of these pathogenic autoantibodies and excess cytokines through plasma exchange directly halts this lytic cascade on the capillary and glomerular endothelium (15, 16).

### 3.2 Atypical case presentation

The main diagnostic dilemma and the most atypical aspect of this case lay in its initial clinical presentation. The triad of acute respiratory failure, bilateral alveolar infiltrates, and critically elevated acute-phase reactants guided the initial algorithm toward severe bacterial pneumonia complicated by acute respiratory distress syndrome. In addition, the gastrointestinal debut symptoms (profuse watery diarrhea and vomiting) mimicked a systemic infectious process or sepsis of enteral origin. The literature describes that a minority of patients with reactivation of ANCA-associated vasculitis may present with aberrant constitutional or gastrointestinal symptoms due to vasculitis ischemia in the intestinal mucosa, which often masks the systemic disease and delays the correct diagnosis (17, 18).

The decisive factor that reoriented the clinical suspicion within the first 48 to 72 h was the finding of rapidly developing progressive anemia without obvious external bleeding, accompanied by rapidly progressive renal dysfunction on a baseline chronic nephropathy (18). Quick exclusion of differential diagnoses through biomarkers and immunoserology avoided invasive procedures (such as bronchoalveolar lavage or biopsy) in a critically unstable patient. Unlike Goodpasture syndrome (anti-GBM disease) or lupus nephritis, which have specific immune profiles and, in the case of lupus, severe hypocomplementemia due to immune complex deposition, this patient maintained strictly normal C3 and C4 levels, negativity for anti-GBM antibodies, ANA, and anti-DNA, combined with a critically high and isolated MPO-ANCA peak (514.80 CU) (14, 19).

Additionally, the persistence of low procalcitonin levels (0.42 ng/mL) and negative blood cultures allowed the case to be classified as a primary autoimmune crisis and to rule out concomitant bacterial sepsis at onset, given the high negative predictive value of procalcitonin for distinguishing vasculitic activity from systemic bacterial infection (20). The subsequent isolation of ESBL-producing *Escherichia coli* in the urinary tract was ultimately interpreted as a focal, nosocomial infectious complication and not as the trigger of the pulmonary collapse.

### 3.3 Diagnostic approach

On admission, the presence of hypoxemic acute respiratory failure, bilateral alveolar infiltrates, gastrointestinal symptoms (diarrhea and vomiting), and marked elevation of acute-phase reactants required a thorough and systematic diagnostic algorithm. The initial approach proposed severe pneumonia complicated by acute respiratory distress syndrome secondary to a systemic infectious process or sepsis. However, a multi-disciplinary diagnostic evaluation during the first 48–72 h allowed the exclusion of alternative causes of acute respiratory distress syndrome and unequivocally confirmed a reactivation of MPO-ANCA-associated vasculitis with diffuse alveolar hemorrhage.

To rule out cardiogenic pulmonary edema or volume overload as the origin of infiltrates, a bedside transthoracic echocardiography (*Point-of-Care Ultrasound*, POCUS) was immediately performed after admission. This showed a preserved left ventricular ejection fraction (60%), normal segmental contractility, absence of hemodynamically significant valvular disease, and an inferior vena cava diameter of 16 mm with inspiratory collapsibility greater than 50%. These findings correlated with B-type natriuretic peptide levels of 112 pg./mL (slightly modified by renal dysfunction), ruling out a cardiogenic etiology or elevated left ventricular filling pressures. Also, given the risk of pulmonary thromboembolism in a critically pro-inflammatory state, the angiographic phase of the chest CT scan was thoroughly analyzed, ruling out intraluminal filling defects in the main pulmonary artery and its lobar, segmental, or subsegmental branches.

Due to the patient’s extreme ventilatory instability (PaO_2_/FiO_2_ = 58) under protective mechanical ventilation parameters with elevated positive end-expiratory pressures (>12 cm H_2_O), bronchoscopy with bronchoalveolar lavage was formally contraindicated due to the prohibitive risk of barotrauma, critical alveolar recruitment, or exacerbation of microvascular bleeding. In the absence of endoscopic confirmation, the diagnosis of diffuse alveolar hemorrhage was established by the following objective and concurrent diagnostic evidence: 1) manifest hemorrhage, with serial endotracheal aspirates persistently frankly bloody through the orotracheal tube, ruling out epistaxis or upper airway bleeding; 2) prior hemoptysis, indirect questioning of relatives confirmed episodes of cough with frank hemoptysis in the 6 h prior to respiratory collapse; 3) hemoglobin kinetics, drop from an outpatient baseline documented at 14.5 g/dL to 8.9 g/dL on admission, reaching a critical nadir of **7.7** g/dL in the first 48 h, without evidence of gastrointestinal bleeding (negative rectal exam and nasogastric lavage) or other external blood loss; 4) tomographic distribution, classic CT pattern of bilateral ground-glass opacities and small perihilar and central consolidations with relative preservation of the subpleural pulmonary cortex (Figure 1A).

Given the profound immunosuppression secondary to previous exposure to cyclophosphamide (6-month induction in 2024), cumulative rituximab doses (2 g in June 2025), and maintenance corticosteroid therapy (prednisone 20 mg/day), a comprehensive microbiological panel was structured to rule out viral, fungal, and opportunistic infections: a multiplex real-time PCR molecular panel on nasopharyngeal and tracheal secretions was negative for SARS-CoV-2, Influenza A (including H1N1 and H3N2 subtypes), Influenza B, and Respiratory Syncytial Virus; *Pneumocystis jirovecii* was ruled out by a negative real-time PCR in the tracheal aspirate. Quantitative viral load for cytomegalovirus by serum PCR was undetectable, excluding significant viremia. Urinary antigen for *Streptococcus pneumoniae* and *Legionella pneumophila* was negative. Repeated bacterial and fungal cultures of tracheal aspirate and serum galactomannan antigen did not show pathogen growth or invasive mycoses. The later identification of a urinary tract infection caused by *Escherichia coli* ESBL was treated appropriately and cataloged as a focal, confined infectious complication that did not explain the pathophysiology of the pulmonary collapse.

The patient had baseline impaired renal function secondary to previous renal involvement from vasculitis in 2024, with a mean baseline serum creatinine of 1.40 mg/dL; estimated glomerular filtration rate [eGFR] by CKD-EPI of approximately 41 mL/min/1.73m^2^. During the current presentation, creatinine acutely increased to 2.19 mg/dL on admission and rapidly escalated to 2.61 mg/dL (with a maximum hospital peak of 3.07 mg/dL), confirming acute kidney injury KDIGO stage 3B superimposed on her chronic kidney disease.

At the last outpatient follow-up in remission (2025), MPO-ANCA titers had remained stable at subclinical ranges. However, coinciding with the current presentation, anti-myeloperoxidase antibodies (MPO-ANCA) showed a critical exponential rise, reaching a titer of 514.80 CU (chemiluminescent units), while anti-proteinase 3 antibodies (PR3-ANCA) remained undetectable (<2.3 CU). This drastic elevation of autoantibodies, together with worsening 24-h proteinuria (increasing from her chronic baseline of 3 g to a peak of 4035.36 mg), provided definitive evidence of systemic immunological reactivation. Additionally, normal complement C3 (156.2 mg/dL) and C4 (35.7 mg/dL) fractions, along with absolute negativity for antinuclear antibodies (ANA), double-stranded anti-DNA, and the ENA profile, excluded immune complex-mediated glomerulonephritis such as lupus nephritis.

### 3.4 Therapeutic intervention

After admission to the intensive care unit, the patient developed hypoxemic respiratory failure secondary to diffuse alveolar hemorrhage, requiring orotracheal intubation and initiation of protective mechanical ventilation. Because severe concomitant pneumonia could not be completely ruled out on admission, empiric broad-spectrum antibiotic therapy was started while microbiological studies were performed. At the same time, the suspicion of MPO-ANCA-associated vasculitis relapse—supported by clinical deterioration, increased MPO-ANCA titers (514.8 CU), increased acute-phase reactants (CRP 186 mg/L), and worsening renal function—led to the immediate initiation of intravenous methylprednisolone pulses (500 mg/day for 3 days).

As a complementary immunomodulatory measure, intravenous immunoglobulin (2 g/kg) was administered. Although intravenous immunoglobulin is not a standard first-line treatment for severe ANCA-associated vasculitis relapse, in this case it was considered adjunctive therapy due to the coexistence of potentially life-threatening diffuse alveolar hemorrhage and a high initial suspicion of respiratory infection, circumstances in which the aim was to enhance control of the inflammatory response while avoiding immediate intensification of immunosuppression as microbiological evaluation was completed and the patient stabilized. There were no data on significant hypogammaglobulinemia; therefore, its use was based on individualized clinical criteria as a bridging therapy and did not replace conventional immunosuppressive treatment.

During the course in the ICU, initial improvement in oxygenation was observed; however, the patient experienced a new episode of diffuse alveolar hemorrhage with acute respiratory deterioration, resulting in failed extubation, requiring reintubation and subsequently tracheostomy. Given the persistence of severe vasculitic activity, five sessions of therapeutic plasmapheresis were performed. Although the benefit of plasma exchange in ANCA-associated vasculitis remains a subject of debate following the PEXIVAS study, and current recommendations reserve its use for selected situations, in this case the indication was based on the combination of refractory diffuse alveolar hemorrhage with life-threatening risk and acute renal deterioration on chronic kidney disease (maximum creatinine 3.07 mg/dL), scenarios in which some centers still consider plasma exchange as a rescue therapy.

After completing the plasmapheresis sessions, the patient achieved clinical stabilization, with no new episodes of alveolar hemorrhage, progressive improvement in oxygenation, decrease in inflammatory markers (CRP from 186 to 24.5 mg/L), and partial recovery of renal function, with serum creatinine decreasing from a maximum of 2.61 to 1.79 mg/dL.

During her stay in the ICU, she developed a urinary tract infection caused by *Escherichia coli* producing extended-spectrum beta-lactamase, which was treated with targeted carbapenems according to the antibiogram. Due to this infectious episode, maintenance therapy with rituximab was temporarily postponed until microbiological control and clinical stabilization were achieved. Once the infection was resolved and there was no evidence of persistent infectious activity, a new dose of rituximab was administered on January 30, 2026, as remission induction treatment, considering the severe relapse of the disease despite prior immunosuppressive treatment and in accordance with current recommendations for ANCA-associated vasculitis. No further episodes of diffuse alveolar hemorrhage were documented thereafter.

Follow-up chest computed tomography, performed 14 days after the initial study, showed marked resolution of the ground-glass opacities and pulmonary consolidations, with only residual interstitial changes and probable chronic centrilobular and paraseptal cystic lesions remaining (Figure 1B). After progressive recovery of respiratory function, the patient was successfully decannulated and discharged from the intensive care unit.

In outpatient follow-up, the patient has remained clinically stable, with no new episodes of diffuse alveolar hemorrhage or evidence of MPO-ANCA-associated vasculitis relapse. She is currently on maintenance therapy with oral mycophenolate mofetil (500 mg every 12 h) and oral prednisone (5 mg/day), as well as prophylaxis against *Pneumocystis jirovecii* with oral cotrimoxazole (800/160 mg three times a week). As part of the management of her comorbidities and chronic kidney disease, she continues treatment with oral enalapril (5 mg/day), amlodipine (5 mg/day), dapagliflozin (10 mg/day), sodium bicarbonate (500 mg/day), and rosuvastatin (20 mg/day). At the last clinical evaluation, the patient maintained respiratory and functional stability, with no need for supplemental oxygen and stable renal function.

The timeline of clinical evolution, therapeutic interventions, and clinical response is detailed in Table 1.

**Table 1:** Chronology of clinical evolution, therapeutic interventions, and clinical response.

Period/date	Clinical phase/Event	Therapeutic interventions	Clinical evolution and response
Year 2024	Onset and diagnosis MPO-ANCA-associated vasculitis with pulmonary and renal involvement.	Induction therapy with cyclophosphamide for 6 months.	Initial remission. Establishes chronic kidney disease with a mean baseline creatinine of 1.40 mg/dL and a glomerular filtration rate of approximately 41 mL/min/1.73 m^2^.
June 2025	Maintenance	Administration of rituximab (cumulative dose of 2 g).	Subsequent outpatient follow-up shows persistent disease activity (proteinuria of 3–4 g/24 h). Receiving prednisone 20 mg/day.
January 2026 *(Emergency Department Admission)*	Acute reactivation and respiratory collapse Progressive dyspnea of 12 h, profuse diarrhea, and vomiting.	• Orotracheal intubation and immediate admission to the ICU. • Initiation of protective mechanical ventilation. • Empiric broad-spectrum antibiotic therapy.	• Severe refractory hypoxemia (SpO₂ 62% on room air). • Blood gas: PaO₂/FiO₂ = 58 (severe ARDS). • Chest CT: Bilateral ground-glass opacities (Diffuse Alveolar Hemorrhage).
First 48–72 h *(In the ICU)*	Confirmation of relapse and multiorgan failure	• IV methylprednisolone pulses (500 mg/day for 3 days). • IV immunoglobulin 2 g/kg (bridging therapy).	• Diagnosis of Diffuse Alveolar Hemorrhage: Confirmed by bloody endotracheal aspirates and a drop in Hb (from a baseline of 14.5 g/dL to a nadir of 7.7 g/dL). • Critical elevation of MPO-ANCA: 514.80 CU and CRP 186 mg/L. • Acute Kidney Injury KDIGO 3B: Creatinine rising from 2.19 to 2.61 mg/dL.
ICU Course *(Subsequent days)*	Refractoriness and complications	• Extubation attempt (failed). • Orotracheal reintubation. • Tracheostomy performed. • Initiation of 5 plasmapheresis sessions (rescue therapy).	• New episode of Diffuse Alveolar Hemorrhage and acute respiratory deterioration. • Maximum in-hospital creatinine peak: 3.07 mg/dL. • Development of a urinary tract infection caused by *E. coli* ESBL (treated with targeted carbapenems; rituximab temporarily postponed).
Post-plasmapheresis	Stabilization and Immunological Control	Completion of plasmapheresis and resolution of the UTI.	• Clinical stabilization with no new bleeding. • CRP decrease from 186 to 24.5 mg/L. • Partial renal recovery (creatinine down to 1.79 mg/dL).
January 30, 2026	Induction Therapy	Administration of a new dose of rituximab after microbiological control.	No further episodes of Diffuse Alveolar Hemorrhage.
14 days post-admission	Radiological Resolution and Weaning	Progressive withdrawal of ventilatory support and successful decannulation.	Follow-up chest CT shows marked resolution of opacities and consolidations.
February 2026 (Approx.)	ICU Discharge and Hospital Discharge	Discharge from the ICU for subsequent medical discharge.	Transfer and discharge with respiratory and functional stability.
July 2026 *(Present)*	Outpatient Follow-up (Remission)	Current treatment: • Mycophenolate mofetil (500 mg every 12 h). • Prednisone (5 mg/day). • Prophylaxis: Cotrimoxazole. • Chronic kidney disease/cardiovascular risk management: Enalapril, amlodipine, dapagliflozin, sodium bicarbonate, and rosuvastatin.	• Clinically stable patient. • No need for supplemental oxygen. • No signs of vasculitis relapse. • Stable renal function.

### 3.5 Patient perspective

The therapeutic success observed in this case highlights the critical role of intensive multimodal intervention through the sequential use of methylprednisolone pulses, intravenous immunoglobulin, and rescue plasmapheresis in the management of refractory diffuse alveolar hemorrhage due to MPO-ANCA vasculitis. Despite the highly recurrent nature and refractory behavior the disease showed after previous regimens with cyclophosphamide and rituximab, the rapid removal of autoantibodies through plasma exchange not only allowed stabilization of the alveolar-capillary barrier and successful weaning from mechanical ventilation, but also induced partial recovery of renal function by mitigating acute inflammatory damage superimposed on baseline chronic nephropathy. However, the persistence of residual interstitial changes and centrilobular cystic lesions on follow-up CT underscores the development of permanent structural sequelae, consolidating the need to reintroduce biological therapy with rituximab to sustain immunological remission.

In the long term, the patient’s prognosis remains conditioned by a delicate balance between the latent risk of severe organ relapses and the extreme vulnerability to opportunistic infectious complications, a challenge exacerbated by the accumulated immunosuppressive burden and prior isolation of *Escherichia coli* ESBL. Likewise, this case vividly illustrates the barriers described in resource-limited settings of the Andean region, where the absence of robust epidemiological records and difficulties in continuous access to specialized biomarkers and advanced induction therapies perpetuate disparities in clinical outcomes. Consequently, the patient’s outlook will depend on strict multidisciplinary follow-up (Rheumatology, Nephrology, and Pulmonology) focused on monitoring renal function, close surveillance of drug toxicity, and early detection of any signs of lupus or vasculitic flare in the outpatient setting.

### 3.6 Limitations

This case also highlights the challenges posed by the management of complex autoimmune diseases in healthcare settings with limited resources, where restricted access to specialized immunological tests, advanced immunotherapies, and multidisciplinary care can delay diagnosis and treatment. In Ecuador, epidemiological data on ANCA-associated vasculitis remain limited, and most information comes from isolated case reports. These limitations may contribute to late diagnosis and worse outcomes in severe cases.

## 4 Conclusion

In this case, the patient developed acute respiratory failure and acute kidney injury consistent with a flare-up of vasculitis. The initial differential diagnosis included severe infectious pneumonia complicated by acute respiratory distress syndrome, particularly given the patient’s immunosuppressed state and elevated inflammatory markers. However, progressive anemia without external bleeding, markedly elevated MPO-ANCA titers, worsening renal function, and the absence of microbiological evidence of systemic infection strongly supported an autoimmune etiology. Furthermore, the clinical presentation was consistent with diffuse alveolar hemorrhage, a recognized life-threatening manifestation of ANCA-associated vasculitis resulting from pulmonary capillary and endothelial injury.

Pulmonary-renal syndrome represents a critical complication of ANCA-associated vasculitis and requires rapid differentiation from other causes, such as glomerular basement membrane disease and severe infections, given that treatment strategies differ substantially. In this case, negative anti-GBM antibody results and elevated MPO-ANCA levels supported the diagnosis of vasculitic reactivation. Early recognition was essential for initiating appropriate immunosuppressive therapy and preventing irreversible organ damage.

The treatment of severe ANCA-associated vasculitis involves intensive immunosuppressive therapy combined with supportive care. Current evidence supports the use of high-dose glucocorticoids and rituximab or cyclophosphamide as induction therapy, especially in cases with severe organ involvement. Plasmapheresis may provide additional benefit in selected patients with diffuse alveolar hemorrhage or rapidly progressive renal failure, particularly when disease activity is refractory to initial treatment. In this patient, intensification of immunomodulatory therapy—which included pulse corticosteroids, plasmapheresis, and rituximab—resulted in the stabilization of respiratory and renal function, underscoring the importance of early and intensive treatment.

The clinical course was further complicated by a healthcare-associated infection caused by ESBL-producing *Escherichia coli*, a known risk in immunocompromised patients receiving intensive immunomodulatory therapy. Despite this complication, specific antimicrobial treatment and ongoing multidisciplinary management contributed to a favorable clinical recovery.

The favorable clinical outcome observed in this patient underscores the importance of early clinical suspicion, immediate immunosuppressive therapy, and coordinated multidisciplinary management involving specialists in critical care, rheumatology, nephrology, and pulmonology. Timely intervention can significantly improve the prognosis, even in patients with severe pulmonary-renal syndrome and multiorgan involvement.

## Data Availability

The original contributions presented in the study are included in the article/supplementary material, further inquiries can be directed to the corresponding author.
